# Variability in feed intake the first days following weaning impacts gastrointestinal tract development, feeding patterns, and growth performance in nursery pigs

**DOI:** 10.1093/jas/skad419

**Published:** 2023-12-23

**Authors:** Lluís Fabà, Tetske G Hulshof, Kelly C M Venrooij, Hubèrt M J Van Hees

**Affiliations:** Trouw Nutrition, Research and Development, Amersfoort, The Netherlands; Trouw Nutrition, Research and Development, Amersfoort, The Netherlands; Trouw Nutrition, Research and Development, Amersfoort, The Netherlands; Trouw Nutrition, Research and Development, Amersfoort, The Netherlands; Faculty of Veterinary Medicine, Department of Veterinary and Biosciences, Ghent University, Merelbeke, Belgium

**Keywords:** biogenic amines, feed intake, growth performance, gut health, histomorphometry, nursery pig

## Abstract

The present study investigated the effects of voluntary feed intake (**FI**) the first days after weaning on gastrointestinal development and protein fermentation the first week after weaning and growth performance and feeding patterns during the nursery phase. A total of 144 mixed-sex weaned pigs (24 ± 2 d old; 7.2 ± 0.8 kg body weight [**BW**]) were allocated to 12 pens with 12 pigs/pen. Each pen was equipped with an electronic feeding station for monitoring individual FI during a 40-d study. Pigs were classified based on their cumulative FI over the initial 3 d after weaning (FId1-3) being above or below their pen median FId1-3 (high = 626 ± 193 g or low = 311 ± 181 g FId1-3). Similarly, weaning BW classes (**BW0**; high = 7.72 ± 0.59 kg or low = 6.62 ± 0.88 kg BW) were created to study interactions with FId1-3. Two female pigs with either a high or a low FId1-3 per pen (*n* = 24) were selected for sampling at d6 and were used to study gastrointestinal development and fermentation products in the small intestine. Feeding patterns per day, FI, and growth performance were measured individually. Low FId1-3 pigs had lower (*P* < 0.05) daily FI during d0 to d8, d8 to d15, and d22 to d28, BW on d15, d22, d29, and d40, and average daily gain during d0 to d8, d22 to d29, and d29 to d40 compared to high FId1-3. High FId1-3 pigs increased (*P* < 0.05) the number of visits to the feeder between d1 to d13 and d31 to d35, and the time spent per visit only for d1 to d4 (*P* < 0.05). The daily rate of FI (g/min) was higher (*P* < 0.05) for High FId1-3 pigs on d6, d8, d9, and d10, and again several days later (d20 to d39). In addition, the high FId1-3 × high BW0 interaction improved daily FI during d18 to d40 compared to low FId1-3 × low BW0 class (*P* < 0.05). For the sampling on d6, low FId1-3 pigs had a lighter small intestine, colon, and pancreas, and reduced villi length, smaller villi surface area, and a lower number of goblet cells size in jejunum (*P* < 0.05), while concentrations of lactic acid, histamine, and cadaverine in small intestinal content were increased (*P* < 0.05). In conclusion, pigs with high FId1-3 became faster eaters with higher FI and growth rates toward the second half of the nursery, which was similar and additive for pigs with higher weaning BW. High FId1-3 was also associated with greater development of the gastrointestinal tract and a reduced protein fermentation 1-wk after weaning.

## Teaser

Early feed intake after weaning was associated with overall feeding patterns and performance, the development of the gastrointestinal tract, and protein fermentation products in the small intestine.

## Introduction

The weaning event and the transition to solid feed remain a challenge to pig husbandry. In effect, it is common that weanling pigs show null or low feed intake (**FI**), as below maintenance energy requirements, for the first 3 to 5 d after weaning (Le Dividich and Herpin, 1994; Bruininx et al., 2001). The lack of FI and, thereby, nutrients in the intestinal lumen results in disturbances including intestinal villus atrophy, reduced enzyme activity and nutrient absorption (Brooks and Tsourgiannis, 2003; Moeser et al., 2017), increased intestinal permeability (Carey et al., 1994), and inflammation (Lallès et al., 2007) which altogether results in a period of lower bodyweight (**BW**) gain (Dong and Pluske, 2007). Furthermore, the presence of unabsorbed nutrients in the lumen, especially protein, causes undesired intestinal fermentation which is widely acknowledged as a risk factor for digestive disorders and disease (Kim et al., 2012; Zhang et al., 2020). Proteolytic fermentation by intestinal microbiota includes relevant end-products such as short-chain and branched-chain fatty acids, ammonia, hydrogen sulfide and methanethiol, phenol and indole compounds, and biogenic amines (Zhang et al., 2020). Importantly, high concentration of biogenic amines in the intestinal lumen have been associated with diarrhea, inflammation, and poor performance in pigs (Pieper et al., 2012; Li et al., 2022). Nonetheless, the associations between poor FI early after weaning, gastrointestinal development, protein digestion, and performance remain largely unknown. This is especially the case under the current worldwide trends of reducing dietary N emissions, and the refraining from antibiotics and ZnO in feed.

Several internal and external factors influence FI immediately after weaning and, thereby, the adaptation to solid feed. Piglet birth weight, general health, stress, growth, and gastrointestinal development prior to weaning, and BW and age at weaning are acknowledged as crucial factors to prepare the pig for overcoming the diet shift (Lallès et al., 2007). Visual sorting of pigs by size as a proxy for weaning BW could be a trait used by producers. Weaning BW is predictor for performance up to slaughter (López-Vergé et al., 2018). However, heavy pigs at weaning may be more susceptible to weaning stressors. In fact, a heavier category of pigs at weaning showed lower initial FI after weaning than their lighter counterparts (Bruininx et al., 2001). Faster-growing piglets during the suckling phase were associated with higher sow milk consumption (Tang et al., 2022); however, when lacking familiarity and interest in solid creep feed, heavy pigs seem more susceptible to weaning stress (Tang et al., 2022). Despite extensive research and a wide array of measures developed to enhance FI early after weaning (Wensley et al., 2021), little is known about the natural variance of immediate after weaning FI within a group of pigs and the reasons driving it. In a recent paper, Engelsmann et al. (2023) reported that individually caged pigs with a higher FI d0 to d4 after weaning had improved overall growth performance and villus surface area with more acid mucus-producing goblet cells (**GC**) but increased concentrations of inflammatory markers, higher risk of diarrhea, and a higher frequency of antibiotic use compared to pigs with lower FI. Thus, a higher FI early after weaning may not be necessarily better for pig health and antibiotic use, while further investigation in this area and group-housing systems is warranted.

We hypothesized that poor FI early after weaning would impair gastrointestinal tract (**GIT**) function with negative effects on growth performance. Furthermore, high vs. low early after weaning FI would be associated with different feeding patterns and interact with weaning BW. Pigs with high weaning BW perform worse short term but better long term in the nursery. Therefore, the present objectives were to characterize pigs with a high or low early FI (d1 to d3) after weaning to study 1) their individual feeding patterns and performance throughout the nursery phase, 2) their interaction with high and low weaning BW, and 3) their effect on GIT organ weights, histomorphometry, and protein digestion the first week after weaning.

## Material and Methods

### Ethical approval

The internal animal welfare body of Trouw Nutrition R&D reviewed and approved the experimental protocol and followed the Central Committee Animal Experimentation (CCD, The Hague, The Netherlands) approval with animal use protocol no. AVD2040020184665.

### Animals, housing, and experimental design

A total of 144 mixed-sex pigs (Hypor Libra × Hypor Maxter, Hendrix Genetics B.V., Boxmeer, The Netherlands) were used in a 40-d study at the Trouw Nutrition R&D Swine Research Centre (St. Anthonis, The Netherlands). Pigs were selected from 20 litters excluding pigs that previously received medication or had poor growth during the suckling phase. From d5 all litters were offered a piglet milk replacer (i.e., crude protein [**CP**] = 20%, ether extract [**EE**] = 15%, lactose = 36%, net energy [**NE**] = 13.8 MJ/kg, standardized ileal digestible (**SID**) Lys = 16 g/kg; Milkiwean Babymilk; Trouw Nutrition Nutreco Belgium NV, Ghent, Belgium) and from d12 onwards they were offered a standard commercial creep feed (i.e., CP = 19%, EE = 10%, lactose = 15%, NE = 11.7 kcal/kg, SID Lys = 14 g/kg; Milkiwean Precoce; Trouw Nutrition Nutreco Belgium NV, Ghent, Belgium) until weaning. Pigs were weaned at 24 ± 2 d of age (7.16 ± 0.80 kg BW) and allocated to two rooms with 6 pens/room and 12 pigs/pen. The allocation was based on BW at weaning (BW d0), litter origin, and sex with 5 males and 7 females per pen. Each pen (3.0 m × 1.67 m) was equipped with an electronic feeding station (**EFS**, Schauer Agrotronic, GmbH, Austria) having 15 cm feeder space. The EFS was activated by the pig’s ear tag Radio-Frequency IDentification (RFID, MS Tag Round HDX STF-YELOPPRPB1, MS Schippers BV, Hapert, The Netherlands) enabling monitoring individual FI and feeding pattern. Room lighting and EFS lights were switched on between 0600 and 2200 hours and temperature was set at 30 °C at weaning and gradually reduced to 25 °C on d28 until the end of the nursery.

FI classes were defined based on cumulative FI over the initial 3 d (FId1-3) after weaning. The FId1-3 criterion was chosen based on literature (Le Dividich and Herpin, 1994; Bruininx et al., 2001) as having negative effects on the GIT and performance (Spreeuwenberg et al., 2001; Engelsmann et al., 2023). Within each pen, pigs were classified for FId1-3 as above (High *n* = 58; 626 ± 193 g FId1-3) or below (Low *n* = 53; 311 ± 181 g FId1-3) their pen median FId1-3 after removing the two pigs per pen that were selected for euthanasia and sampling on d6 (see below) and other removed pigs. The FI on d0 was not recorded because the EFS was continuously open without ear tag registration for adaptation. Besides the FId1-3 class, a similar classification was created based on weaning BW, and pigs were classified as High (*n* = 56; 7.72 ± 0.59 kg BW) or Low weaning BW (*n* = 55; 6.62 ± 0.88 kg BW) relative to their pen median for further comparison. The choice for studying average high and low BW classes instead of four quartiles was made 1) to evaluate if being an average heavy or a light pig was already meaningful in its interacting with initial FI following weaning, and 2) so equivalent BW distribution was present within FId1-3 classes.

At d 6 after weaning, two female pigs per pen (*n* = 24 in total) were selected for euthanasia and tissue sampling. One female pig per pen had a High FId1-3 and the other female pig per pen a Low FId1-3 (highest and lowest in the pen, respectively) to study the effect of initial FI on GIT, including organ weights, jejunum histomorphometry, small intestine fermentation products and CP content as detailed later at sample collection. Only females were selected to reduce variation and minimize sample size since females showed more adverse responses in GIT development caused by weaning stress than their male counterparts (Pluske et al., 2019).

Two experimental diets were formulated and produced at an experimental feed plant (ForFarmers, Heijen, The Netherlands) and were fed during d0 to d15 and d15 to d40, respectively, to all pigs. Diets were a wheat–barley–soybean meal-based diets formulated to meet or exceed the nutrient requirement of weanling pigs (NRC, 2012). During the entire period, pigs had ad libitum access to feed and water (one nipple per pen).

### FI, feeding patterns, and growth performance

The EFS recorded feed disappearance and start and end time of each visit per pig using a RFID tag. Failed identification or incorrect recordings of feed disappearance are main sources of errors in the data collected by EFS (Eissen et al., 1998). Therefore, feed disappearance data per visit were screened before further calculations of feeding patterns as proposed by Bruininx et al. (2001, 2003). Furthermore, weekly manual weighing of left-over feed per pen was compared to the EFS raw data to ensure adequate functioning of the EFS as per standard operational procedures. The data curation process allowed for individual monitoring of FI pattern variables. Data included the daily number of visits to the trough (we only report visits with feed disappearance > 0 g), time per visit, and amount consumed per visit from d1 after weaning onwards. Variables were used to calculate FI per day and the rate of FI as FI per visit divided by visit time per day (in g/min).

Individual BW was determined on d0, d8, d15, d22, d29, and d40, and average daily gain (**ADG**), average daily FI (**ADFI**) and feed efficiency (**FE**; as ADG:ADFI) were calculated for the corresponding period, i.e., d0 to d8, d8 to d15, d15 to d22, d22 to d29, and d29 to d40. In case of mortality or culling, the animal was weighed, and the reasons recorded.

Diets were quality checked by analyzing moisture (EC regulation 152/2009, appendix III A), nitrogen (N; ISO 16634-1:2008) and CP calculated as N × 6.25, acid hydrolyzed EE (EC regulation 152/2009, appendix III H method A), crude fiber (ISO 6865:2000), acid insoluble ash (EC regulation 152/2009, appendix III N), Lys (NEN-EN-ISO 13903), and Ca, P, Na, and Zn by ICP-OES spectrometry (Perkin-Elmer S10, model Avio 200; MA; ISO 15510:2008).

### Sample collection

The 24 female pigs that were selected for euthanasia and tissue sampling on d6 were sedated using a mixture of Zoletil (250 mg zolazepam and 250 mg tiletamine; VIRBAC, Carros, France) and 20 mL Sedanum (20 mg xylazine/mL; Dechra Pharmaceuticals, Northwich, United Kingdom) at 1 mL per 10 kg BW and, thereafter, humanly euthanized by intracardiac injection with 40% barbiturate pentobarbital (390 mg pentobarbital sodium and 50 mg phenytoin sodium per mL, Euthasol, Virbac, Carros, France).

At dissection, measurements included BW, organ weights including pancreas, liver, and for the GIT sections full and empty weights of the stomach, small intestine, cecum, and colon including the rectum. The content weights were calculated by difference and the weights from the empty organs and their contents were expressed as g/kg BW at euthanasia. A homogeneous 50 g content sample from the stomach, pooled together small intestine sections (duodenum, jejunum, and ileum content), and pooled content from the colon were collected, snap frozen on dry ice, and stored at −20 °C until further analysis. The pH was determined in the contents of the stomach, pooled together small intestine content, and colon (pH meter WTW 3110 ProfiLine, 2023 Thermo Fisher Scientific Inc., Waltham, MA). Furthermore, a 3-cm tissue sample from jejunum was obtained from 30 cm caudal to the pylorus and fixed in a cassette including 4% buffered formaldehyde for histomorphometry and GC counting and measuring by the laboratory GD animal health (Royal GD, Deventer, The Netherlands).

### Histomorphometry and GC in jejunum

After arrival of the tissue samples at the laboratory, they were dehydrated and embedded in paraffin. Tissue blocks were sectioned at 2 μm, mounted on a glass slide, and stained with the combined Alcian Blue (pH 2.5)/PAS procedure. For each batch of slides stained, an Alcian Blue/PAS-positive control slide was included. Microscopic images of representative cross-sections of each tissue were captured by a microscope (Olympus BX41) connected to a digital camera (Olympus Dp26) and analyzed using Olympus cellSens Dimension version 1.12 software. Of each sample, 10 randomly selected well-oriented intact paired villus–crypt units were measured. Villus length (axis top to villus-crypt junction) and crypt depth (from villus-crypt junction to the base of villus) were used to calculate villus length to crypt depth ratio. Goblet cell size and counts of total GC and neutral (PAS positive) and acid mucin (Alcian Blue positive) producing GC were determined and analyzed in five representative well-oriented villi. The GC size and number were reported in μm^2^, as area per villus surface area (in μm^2^), and as percentage of the villus surface area. The average of each villus:crypt ratio, and the total, neutral, and acid mucin GC characteristics were calculated and reported per animal. The coefficient of variation (CV, %) for each variable was calculated on animal level with five measurements per animal. All morphometric measurements were performed by a European College of Veterinary Pathologists board-certified veterinary pathologist.

### Fermentation products in small intestine

The content of volatile fatty acids (**VFA**) and other fermentation products in whole small intestinal pooled contents was analyzed including acetic, butyric, propionic, succinic, valeric, lactic, iso-butyric, and iso-valeric acid. Analyses were conducted at MasterLab (Boxmeer, The Netherlands) as described elsewhere (Van Hees et al., 2019). Briefly, 1 g of homogeneous digesta per sample was diluted with 2 M sulfuric acid, thoroughly mixed and centrifuged. The supernatant was analyzed for VFA by HPLC on a BioRad Aminex HPX-87H using a 0.005 mol/L sulfuric acid eluent at a flow rate of 0.7 mL/min.

Biogenic amines contents, i.e., cadaverine, histamine, putrescine, spermidine, spermine, and tyramine, in small intestinal content were analyzed according to Rosier and Van Peteghem (1988). In brief, a 2 g homogeneous sample was extracted by adding 10 mL trichloric acetic acid 5%, and derivatized with dansyl chloride and NaOH. After derivatization, 0.1 mL extract was mixed with 5 mL cyclohexane hexane. After vortexing and centrifuging (180 × *g* for 5 min at 20 °C), the cyclohexane layer was collected, dried using N^2^ at 45 °C, and resuspended in 1000 μL methanol/acetonitrile (50:50). Thereafter, the extract was analyzed using a Spherisorb C8, 5 µm 250 × 4.6 mm (BGB Analytik, Harderwijk, the Netherlands) on a Thermo Quantis liquid chromatography–tandem mass spectrometer (Heated Electrospray Ionization source) with an Ultimate 3000 pump and auto-sampler (Thermo Fisher Scientific). The elution buffer was a gradient with acetonitrile, water, and formic acid (0.3%).

Some analysis was below detection limit (**BDL**, i.e., <10 mg/L for VFA and < 100 μg/kg for biogenic amines). Data for butyric, iso-butyric, propionic, valeric, and iso-valeric acid were BDL in all samples and therefore were not reported. For the other VFA, in case some samples were BDL but not all, the detection limit value was assigned to the BDL samples to run statistical analysis adequately. Acetic acid and succinic were given a value of 8 mg/L in *n* = 3 and *n* = 14 samples, respectively. Tyramine had *n* = 20 BDL (out of *n* = 24 in total) and data was, therefore, not reported as it could not be statistically analyzed.

### CP and lysine content

The first intention was to conduct CP and Lys digestibility measurements from ileum content but digesta amounts were insufficient for sample processing and analysis in several pigs, likely because of Low FId1-3 selection criteria. In situ, it was decided to pool the content of the small intestine sections as a whole sample per pig (amounts ranged between 25 and 200 g of fresh sample). Therefore, digestibility values are not reported. Small intestine and colon content samples were dried at 70 °C to constant weight in a Memmert oven (Memmert GmbH) to obtain the dry matter concentration (Masterlab, Boxmeer, the Netherlands). After drying, the samples were milled on a Retsch ultra-centrifugal mill with a 0.75-mm sieve. CP was analyzed as nitrogen (N) by the combustion method (method 990.03; LECO FP 528 MI, USA) using the LECO Nitrogen analyzer and CP was calculated as N × 6.25 as in feed. Insoluble ash was measured as in feed (EC regulation 152/2009, appendix III N). The amino acid analyzer was used to determine the total Lys content in digesta according to an in-house method (Nutreco-MasterLab, Boxmeer, Netherlands) based on NEN-EN-ISO 13903. Briefly, a 0.5- to 1-g dried sample was hydrolyzed with a HCl solution and then evaporated into a film evaporator before being taken up by a buffer solution. Samples were then separated on a cation exchanger with a gradient step and postcolumn derivatization with ninhydrin; Lys was then measured at 440 and 570 nm using a flow colorimeter. All samples were analyzed in duplicate on a BIOCHROM 30 + amino acid analyzer.

### Statistical analysis

The normality of data was checked by visual assessment of residual plots and PROC UNIVARIATE (SAS Inst. Inc., Cary, NC). The PROC GLIMMIX procedure was used to analyze growth performance, feeding patterns, and dissection data with dist = gaussian (normally distributed data) link = identity or dist = gamma link = log (nonnormal data i.e., biogenic amines). The statistical model for growth performance and EFS data included the fixed effects of FId1-3 class (High or Low), time (d) and their three-way interaction, and as random effects the intercept (subject = animal) as repeated factor and the pen. The weaning BW was retained as a covariate when *P* < 0.10. Furthermore, to compare and visualize the effects of weaning BW the model was repeated including the weaning BW class (High or Low) and interactions. For the dissection data, the models included FId1-3 class as a fixed effect and pen and room as random effects. The mean separation was done by the PDIFF option and adjusted by the Tukey–Kramer test. The PROC PLM procedure with Slice option was used to report the fixed effect *P* values within time interaction. The experimental unit was the pig. The *P* values < 0.05 were considered significant for all data, and *P* ≤ 0.10 were considered trends.

In addition, PROC REG was used to describe linear relationship and coefficient of determination between FId1-3 and overall ADG and ADFI variables.

## Results

The diets were in accordance with the formulated values except for Lys in phase 1 diet which was 1.1 g/kg below the calculated value (Supplementary Table S1).

### General performance traits

In accordance with our internal standard operation procedure, nine pigs were removed from the study since they had poor adaptation after weaning with less than 80 g FI for the first 3d. In total, three pigs died, and 21 pigs required antibiotic medication including *n* = 12 (22.6%) from Low FId1-3 and *n* = 9 (15.5%) from High FId1-3 (*P* = 0.338).

**Figure 1.**
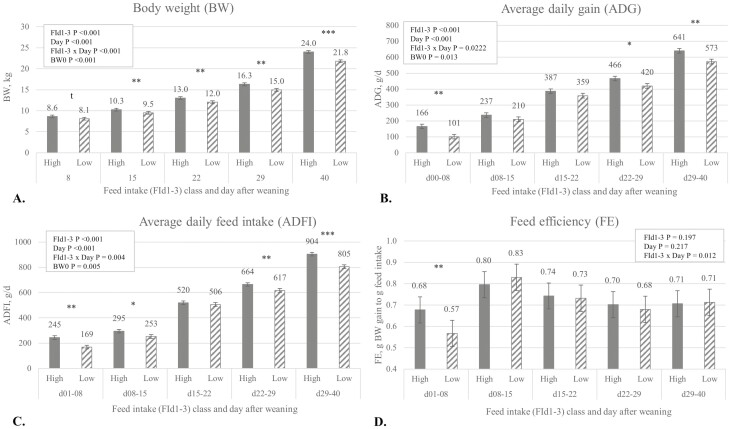
Effect of FI d1 to d3 class (FId1-3) × day interaction on general performance^1^. ^1^Data presents LSMEANS ± SE for pigs from FId1-3 class as above (High, *n* = 58) or below (Low, *n* = 53) their pen (*n* = 12 pens) median for FId1-3.

Pigs classified as above (High) or below (Low) their pen median for FId1-3 had a cumulative FId1-3 intake of 626 ± 193 g and 311 ± 181 g, respectively. Data for growth performance are reported in Figure 1A to D showing significant interactions of FId1-3 with time (*P* < 0.05). The BW tended to be lower on d8 (*P* = 0.062) and was significantly lower for the subsequent days (all *P* < 0.01) for the Low FId1-3 pigs compared to High FId1-3 pigs. In effect, the BW difference between classes became larger over time ranging from 6.2% difference on d8 to 9.2% on d40. For ADG, ADFI, and FE, class × time interactions (*P* < 0.05) showed the loss of a significant effect from FId1-3 class during some periods. Low FId1-3 pigs had reduced ADG during period d0 to d8 (*P* < 0.01), d22 to d29 (*P* = 0.016), and d29 to d40 (*P* < 0.001) after weaning compared to High FId1-3 pigs but not for d8-22 (*P* > 0.140). The ADFI was lower for the Low FId1-3 pigs during d1 to d8 (*P* < 0.001), d8 to d15 (*P* = 0.044), d22 to d29 (*P* = 0.025), and d29 to d40 (*P* < 0.001) compared to High FId1-3 pigs but not for d14 to d22 (*P* = 0.50). In general, for every gram increase in FId1-3, overall ADG increased 0.087 g/d (*P* = 0.01; *R*^2^ = 0.20) and ADFI increased with 0.131 g/d (*P* = 0.01; *R*^2^ = 0.23). There was no significant correlation between BW0 and FId1-3 (*P* = 0.578; *R*^2^ = 0.01). Finally, FE interaction with time (*P* = 0.012) showed that Low FId1-3 pigs had lower FE during d1 to d8 (*P* < 0.01) but not for the other time intervals.

### Feeding patterns

**Figure 2.**
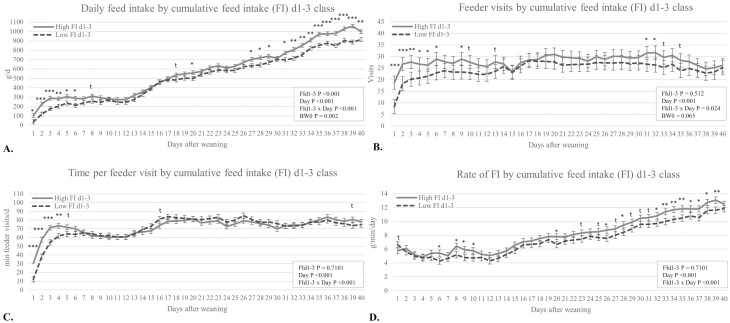
Effect of FI d1 to d3 class (FId1-3) × day interaction on FI (A), number of visits (B), average time spent per visit to the feeder (C), and the rate of FI (D) per day^1^. ^1^Data presents LSMEANS ± SE for pigs from FId1-3 class as above (High, *n* = 58) or below (Low, *n* = 53) their pen (*n* = 12 pens) median for FId1-3.

In Figure 2, the data from the EFS are reported as FI (A), number of visits (B), average time spent per visit (C), and the rate of FI (D) per day. Different feeding patterns throughout the nursery phase were observed between High and Low FId1-3 pigs. High FId1-3 pigs had significantly higher daily FI later in the nursery, i.e., d20, d27 to d29, and d31 to d40 (*P* < 0.05; Figure 2A). High FId1-3 pigs increased (*P* < 0.05) or tended to increase (*P* ≤ 0.10) the number of visits to the feeder (five visits per day more on average) on several days after weaning, i.e., between d1 and d13 (d1 to d6, d9, d10, and d13) and later in the nursery, i.e., between d31 and d35 (Figure 2B). On the other hand, the time spent per visit was only significantly higher for High FId1-3 pigs on d1 to d4 (*P* < 0.05) and tended to be higher on d5 and d39 (*P* ≤ 0.10), while Low FId1-3 pigs tended to spend more time per visit on d16 (*P* ≤ 0.10; Figure 2C). The daily rate of FI was higher for High FId1-3 pigs early after weaning, i.e., on d6, d8, d9, and d10, and again later in the nursery with several occasions being significantly higher (11 d) or showing trends (6 d) between d20 and d39.

Four periods with different daily FI patterns could be distinguished during the nursery phase (Figure 2A). The first week after weaning, i.e., d1 to d8, showed clear differences in FI matching the FId1-3 selection criteria. Afterward, a plateauing FI period d9 to 14 followed by a steady increase of FI for d14 to d26 for both FI classes. Finally, for d27 to d40, pigs from High FId1-3 class had increasing daily FI of around 100 g/d higher than pigs from Low FId1-3 class.

### Early FI × weaning BW

Pigs classified as above (High) or below (Low) their pen median weighed 7.72 ± 0.59 kg and 6.62 ± 0.88 kg BW at weaning, respectively. When studying the FId1-3 × weaning BW interaction for overall performance traits (Table 1), no differences were observed (*P* > 0.05). The BW0 class had no effect on cumulative FId1-3. However, High FId1-3 pigs were 0.2 kg heavier at weaning (*P* = 0.05, SEM = 0.11) than Low FId1-3 pigs (Table 1). High FId1-3 and High BW0 pigs, both showed increased performance including higher final BW (d40), ADG d0 to d40, and ADFI d1 to d40 (*P* < 0.01). The FE d0 to d40 was not affected by FId1-3 or BW0 class (*P* > 0.05).

**Table 1.** Effects of FI d1 to d3 class (FId1-3) and weaning body weight class (BW0) on overall nursery performance^1^^,^^2^

		*N*	FI d1 to d3, g	BW d0, kg	BW d40, kg	ADG d0 to d40, g/d	ADFI d0 to d40, g/d	FE d0 to d40, g/d
FId1-3	High	58	626	7.26	24.0	400	573	0.70
	Low	53	311	7.06	21.8	351	512	0.69
	SEM		76.4	0.111	0.37	8.45	11.28	0.011
	*P* value		<0.001	0.049	<0.001	<0.001	0.001	0.287
BW0 class	High	56	464	7.70	24.2	394	565	0.70
	Low	55	474	6.62	21.6	357	520	0.69
	SEM		76.6	0.110	0.36	10.38	10.86	0.012
	*P* value		0.377	<0.001	<0.001	<0.001	0.004	0.142
FId1-3 × BW0 means								
High FId1-3	High BW0	34	637	7.82	25.1	414	595	0.70
	Low BW0	24	616	6.71	22.9	385	552	0.70
Low FId1-3	High BW0	22	291	7.58	23.3	374	536	0.70
	Low BW0	31	332	6.54	20.3	328	488	0.68

^1^Data LSMEANS of individual pigs per FId1-3 class as above or below their pen (*n* = 12 pens) median for FId1-3 and BW0 class. The means for FId1-3 × BW0 interaction were reported as important information but the interaction with *P* > 0.10 was removed from the statistical model.

^2^FI = feed intake, BW = body weight, ADG = average daily gain, ADFI = average daily FI, FE = feed efficiency (i.e, ADG/ADFI), SEM = standard error of means.

^a,b^LSMEANS within a row without a common superscript differ at *P* < 0.05.

^x,y^LSMEANS within a row without a common superscript tend to differ at *P* ≤ 0.10.

**Figure 3.**
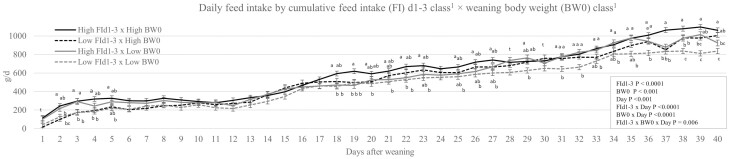
FI d1 to d3 class (FId1-3) × weaning body weight class (BW0) × day interaction on daily FI^1^. ^1^Data LSMEANS ± standard error of individual pigs per FId1-3 class as above or below their pen (n = 12 pens) median for FId1-3 (High, *n* = 58, and Low, *n* = 53) and for BW0 class (High, *n* = 56, and Low, *n* = 55).

The data for daily FI and the interaction between FId1-3 × BW0 class × day are reported in Figure 3. Early after weaning, BW0 alone did not affect (*P* > 0.05) daily FI. The FId1-3 alone explained the observed higher daily FI during d1 to d5. From d18 onwards, BW0 class significantly affected FI with higher intakes for High BW0 pigs. However, for most of these days the effect appeared as an interaction. For d18 to d40, High FId1-3 and High BW0 pigs had higher daily FI compared to Low FId1-3 and Low BW0 pigs. High FId1-3 and Low BW0 pigs and Low FId1-3 and High BW0 pigs were often intermediate and not significantly different from each other. High FId1-3 with Low BW0 and Low FId1-3 with High BW0 were not different from low FId1-3 with Low BW0 for d29, d33, d35, d40 (*P* > 0.05).

### Gastrointestinal tract development

The general results of the pigs selected for euthanasia and sampling are reported in Table 2. Pigs from the Low FId1-3 class showed lower FI 24 h prior to euthanasia than the High FId13 class (*P* = 0.035), however, there were no differences in FI 2 to 12 h prior to euthanasia (*P* > 0.05). Regarding the GIT measurements, the relative weight of (in g/kg BW) the small intestine (*P* < 0.001), colon plus rectum (*P* = 0.021), and pancreas (*P* = 0.028) were lower for Low FId1-3 pigs compared to High FId1-3 pigs. Other organ weights and digesta content weights were not different between FId1-3 classes (*P* > 0.05).

**Table 2.** General traits and gastrointestinal measurements for pigs selected based on FI d1 to d3 (FId1-3)^1^

	FI d1 to d3 class			
	High	Low	SEM^2^	*P* values
	*N* = 12	*N* = 12		
Body weight dissection, kg	8.32	7.76	0.29	0.135
Body weight weaning, kg	7.05	7.25	0.25	0.525
Cumulative feed intake d1 to d3, g	783^a^	236^b^	58	<0.001
Average daily feed intake d0 to d6, g/d	261^a^	124^b^	13.2	<0.001
Average daily gain d0 to d6, g/d	211^a^	85^b^	14.9	<0.001
Feed intake hours prior euthanasia, g				
2h, g	41.5	37.8	8.96	0.726
8h, g	82.8	79.1	11.5	0.788
12h, g	114	102	16.3	0.529
24h, g	326^a^	251^b^	30.2	0.035
Organ weights, g/kg BW				
Stomach weight	8.37	8.6	0.34	0.503
Stomach content	24.7	29.5	2.99	0.276
Small intestine weight	35.5^a^	28.8^b^	1.28	<0.001
Small intestine content	19.6	17.2	1.70	0.337
Cecum weight	1.68	1.40	0.13	0.159
Cecum content	4.73	4.01	0.68	0.455
Colon + rectum weight	14.9^a^	12.9^b^	0.6	0.021
Colon + rectum content	16.4	15.5	1.38	0.599
Pancreas	1.77^a^	1.55^b^	0.07	0.028
Liver	26.1	26.0	0.76	0.931

^1^Data LSMEANS of individual female pigs per FId1-3 class as highest or lowest FId1-3 per pen (*n* = 12 pens).

^2^Standard error of the mean.

^a,b^LSMEANS within a row without a common superscript differ at *P* < 0.05.

Data from jejunum histomorphometry and GC measurements are reported in Table 3. Low FId1-3 pigs had reduced villus length (*P* = 0.004), villus surface area (*P* = 0.043), and a tendency for reduced crypt depth (*P* = 0.056). In addition, Low FId1-3 pigs had an increased variability (CV %) in GC (*P* = 0.026) and acid mucus GC (*P* = 0.042) surface area per villus as percentage of total villus surface area and reduced size of acid mucus GC (*P* = 0.029). There was no effect of FId1-3 class on neutral mucus GC (*P* > 0.05).

**Table 3.** Jejunum histomorphometry and mucus-producing goblet cells measurements for pigs selected based FI d1 to d3 (FId1-3)^1^

	FI d1 to d3 class			
	High	Low	SEM^2^	*P* values
	*N* = 12	*N* = 12		
Villus length, μm	420^a^	345^b^	16.2	0.004
Crypt depth, μm	212^x^	190^y^	7.73	0.056
Villus length to crypt depth ratio	2.08	1.89	0.10	0.135
Surface area villi, mm^2^	40.6^a^	33.1^b^	2.47	0.043
Goblet cells (GC)				
Per villus, *n*	38.1	33.1	5.00	0.484
Size, μm^2^	23.8	19.9	1.99	0.962
Surface area per villus, μm^2^	892	692	150	0.308
Surface area per villus, CV^3^%	31.4^y^	41.6^x^	3.79	0.071
Surface area per villus, % of total villus surface area	2.27	2.07	0.40	0.620
Surface area per villus % of total villus surface area, CV%	32.4^b^	44.5^a^	3.52	0.026
Acid mucus GC				
per villus, *n*	16.8	15.1	1.64	0.457
per villus, %	49	50.2	5.17	0.817
size, μm^2^	41.3^a^	30.9^b^	3.81	0.029
size, CV%	29.7	22.9	2.74	0.101
surface area per villus, μm^2^	674	487	99.0	0.144
surface area per villus, CV%	33.6^y^	42.8^x^	3.77	0.098
surface area per villus, %	77.5	74.3	4.16	0.552
surface area per villus, % of total villus surface area	1.79	1.47	0.29	0.308
surface area per villus % of total villus surface area CV%	35.6^b^	46.4^a^	3.55	0.042
Neutral mucus (N) GC				
per villus, *n*	21.3	18	3.95	0.531
per villus, %	51	49.8	5.17	0.817
size, μm^2^	8.63	8.54	1.30	0.109
surface area per villus, μm^2^	217	205	68.8	0.905
surface area per villus, %	22.5	25.7	4.16	0.552
surface area per villus, % of total villus surface area	0.49	0.6	0.16	0.583

^1^Data LSMEANS of individual female pigs per FId1-3 class as highest or lowest FId1-3 per pen (*n* = 12 pens).

^2^Standard error of the mean.

^3^The coefficient of variation (CV %) for each variable, calculated on animal level (five measurements per animal) was also analyzed but only reported when *P* < 0.10.

### Protein content and fermentation products

The pH of digesta, CP and Lys content in different GIT sections and the VFA and biogenic amines concentration in small intestinal digesta are presented in Table 4. There were no differences in pH, CP content, and Lys content between FId1-3 classes across GIT sections (*P* > 0.05). Regarding VFA, the Low FId1-3 class increased lactic acid (*P* = 0.045) and tended to increase succinic acid (*P* = 0.085) concentrations in small intestinal contents while acetic acid was not different between FId1-3 classes (*P* = 0.366). For the biogenic amines, cadaverine and histamine significantly increased (*P* = 0.002 and *P* = 0.001, respectively) and putrescine tended to be increased (*P* = 0.075) in Low FId1-3 class compared to High FId1-3 class. Of note, the content of biogenic amines on d6 after weaning was overall highly variable with high CV for all of them, i.e., cadaverine 50%, putrescine 44%, spermidine 42%, spermine 72% and more importantly for histamine 148%.

**Table 4.** The pH, crude protein, and Lys content in different gastrointestinal tract sections, and fermentation products in the small intestine for pigs selected based on FI d1 to d3 (FId1-3)^1^

	FI d1 to d3 class			
	High	Low	SEM	*P* values
	*N* = 12	*N* = 12		
Stomach				
pH	3.9	4.0	0.1	0.502
Small intestine				
pH	6.9	6.7	0.1	0.228
CP, g/100g DM	14.8	14.7	0.29	0.830
Lys, mg/100g DM	217	246	13.7	0.120
Fatty acids, mg/L				
Acetic acid	23.1	29.2	4.70	0.366
Lactic acid	984^b^	1,755^a^	327	0.045
Succinic acid	10.4^y^	14.8^x^	1.89	0.085
Biogenic amines, µg/kg				
Cadaverine	576^b^	989^a^	86.9	0.002
Histamine	6,238^b^	28,034^a^	7178	0.001
Putrescine	1,786^y^	2,437^x^	206	0.075
Spermidine	4,788	5,679	651	0.311
Spermine	915	816	176	0.617
Colon				
pH	6.3	6.3	0.05	0.968
CP, g/100g DM	20.2	20.7	0.70	0.541
Lys, mg/100g DM	646	719	57.5	0.349

^1^Data LSMEANS of individual female pigs per FId1-3 class as highest or lowest FId1-3 per pen (*n* = 12 pens). CP = crude protein; DM = dry matter; Lys = lysine.

^a,b^LSMEANS within a row without a common superscript differ at *P* < 0.05.

^x,y^LSMEANS within a row without a common superscript tend to differ at *P* ≤ 0.10.

## Discussion

The present study demonstrated the negative associations of low FI early after weaning on GIT function, feeding patterns, and growth performance in weanling pigs. Although, arguably, the two weaning BW classes represented a relatively minor difference in weight when compared to commercial conditions (1.08 kg BW difference), the high weaning BW class was found to act additively to high early FI improving performance toward the second half of the nursery.

### Early FI, feeding patterns, and growth performance

The weaning transition-associated stressors are multiple and may reduce performance after weaning (Dong and Pluske, 2007; Moeser et al., 2017; Van Kerschaver et al., 2023). A clear dip was not observed in the present study, but pigs plateaued their FI and ADG between d5 and d14. Intriguingly, FId1-3 or BW0 had no effect on FI during such period nor the recovery from it. However, high FId1-3 pigs reduced FI to a larger extent than their counterparts, which may indicate some temporary negative consequences of high initial FI. As it seems, one or more factors overruled the effects of FId1-3 and BW0 classes while being responsible for the anorexigenic phenotype observed during the plateau period. Beyond altered nutrient intake and GIT functions, which are discussed later, other factors such as the microbiota-gut-brain axis and hypothalamic pituitary adrenal response can influence the voluntary FI and could help to explain the present results (Pelleymounter et al., 2000; Salfen et al., 2003; Leon et al., 2019; Margolis et al., 2021). There is evidence that at weaning, enteric generation of neurons and glial cells (non-neuronal cells) is accompanied by a functional maturation of intestinal neural circuits (Roberts et al., 2007; Koenig et al., 2011). However, the medium-to-long term consequences of disrupted nutrient intake on such microbiota-gut-brain axis maturation are largely unknown and deserve future investigation as these could affect feeding behavior in pigs.

### Feeding patterns

From our FI patterns data, pigs from High FId1-3 were visiting the feeder more often and spent more time per visit resulting in higher FI the first week after weaning, however, the pattern changed over time. The differences in FI mostly disappeared in the following 2 wk and reappeared again toward the second half of the nursery. Part of the FId1-3 effect may result from GIT development that increases growth and, thus, metabolic needs (Quiniou and Noblet, 2012; Li and Patience, 2017). Otherwise, the effect could be associated with a genetic variability in FI (Fàbrega et al., 2003; Gao et al., 2021) or other unknown factors related to dominance, feeding behavior, and learning. Unfortunately, behavior was not evaluated, and thus such discussion is speculative. The number of studies investigating individual FI and FI pattern associations to performance for nursery pigs housed in EFS are scarce (Bruininx et al., 2001, 2002, 2003). Data in grow-finishing pigs indicate that the increasing FI is associated with increasing daily feeding duration in the first few weeks (Hyun and Ellis, 2000; Rauw et al., 2006). However, in time, the number of visits and feeding duration decrease with age whereas FI rate increases (Hall et al., 1999; Hyun and Ellis, 2000; Rauw et al., 2006). This is in contrast with the present study in nursery pigs where the number of feeding visits and time spent feeding were rather constant from d4 onwards. However, in general, grow-finishing pigs achieve higher FI in time through faster feeding rate (Hall et al., 1999; Kavlak and Uimari, 2019) which is in accordance with our general observations and for High FId1-3 pigs. Therefore, for nursery pigs, higher FI seemed to be the consequence of a faster FI rate rather than an effect of number of visits or the time spent per visit.

### Weaning body weight

The effects of FId1-3 and weaning BW were found to be independently but additively improving performance when both were high. The importance of weaning BW is well acknowledged and commonly used as predictor for performance up to slaughter weight (López-Vergé et al., 2018; Montoro et al., 2020). The BW0 classes did not affect FI following weaning (d1 to d3). Contrasting, Bruininx et al. (2001), reported that a class of low weaning BW (6.7 kg BW) like the one in the present study (6.6 kg BW), showed higher initial (24h) FI compared to higher BW counterpart classes (i.e., 7.9 kg and 9.3 kg BW). However, in the same study, the heavier pigs consumed more feed than lighter ones from d8 to the end of the experiment, which is in alignment with our findings from d17 onwards. Bruininx et al. (2001) also reported that lighter pigs had more daily visits and a lower FI per visit the first week after weaning compared to heavier pigs. This contrasts with the present study where no differences between BW0 classes were observed. Probably, differences in classification methodology or genetic progress can explain some of the differences. We classified pigs based on pen median for the FId1-3 and BW0 classes while Bruininx et al. (2001) used the complete batch distribution. Classifying the pigs of our study as high or low FId1-3 across all pens yielded the same interpretation of results (data not shown). The approach chosen here intentionally combined and balanced the pen effect as it is known that group interactions in a pen can influence FI (Figueroa et al., 2013; Laskoski et al., 2021). In general, weaning BW in the present study was relatively high, and a lower weaning BW i.e., <6 kg may have caused greater differences or different results. In the present study, weaning BW ranged from 5.4 to 8.9 kg BW but the BW0 classes high and low were the average within pigs above and below the pen median. Hence, a limitation of current study is that the two BW0 classes were not extreme and possibly do not serve to study the lightest pigs in a batch. Instead of studying the more extreme differences, we demonstrated that an average high or average low BW pig at weaning showed differences in growth performance. This was especially the case when interactions with high and low FId1-3 classes were taken into account. Furthermore, the patterns here are limited to the data collected by the EFS and it is possible that different feeding systems or management factors yield different results (Bruininx et al., 2003; Laskoski et al., 2021). The present study pointed out the relevance of weaning BW and initial FI as independent factors influencing feeding behavior and performance early and late in the nursery phase, and how that had an impact on GIT development.

### Gastrointestinal development and protein fermentation

It is known that a smooth transition to solid feed and adequate FI levels early after weaning help maintain GIT development, support nutrient absorption, and improve growth (Pluske et al., 1997; Moeser et al., 2017), which concurs with the differences observed between High and Low FId1-3 pigs. In effect, the findings for Low FId1-3 pigs on overall performance and their poor GIT development expose negative consequences for both the short- and medium-term, i.e., the first week after weaning and the end of the nursery, respectively. Low FI was associated with detrimental factors for GIT function including higher protein fermentation products in the small intestine (Pieper et al., 2012; Li et al., 2022); a reduced villus surface area in jejunum (Kelly et al., 1991; Pluske et al. 1996; Moeser et al., 2017); a reduced size and higher variability for acid mucus-producing GC (Deplancke and Gaskins, 2001; Engelsmann et al., 2023); and lighter sections of the GIT and pancreas (Owsley et al., 1986; Pluske et al., 2003). In a recent study (Engelsmann et al., 2023), these authors used a similar classification for initial FI after weaning (d0 to d4) and reported an improved growth performance and increased villus surface area with more acid mucus-producing GC in the small intestine at d28 for high FI d0 to d4 pigs compared to their low FI counterparts. Such findings agree with those in the present study where Low FId1-3 pigs had increased variability in acid mucus GC surface area and reduced size of acid mucus GC. In contrast to our findings at d6, the high FI d0 to d4 pigs did not show improvements on small intestine villus length at d28 (Engelsmann et al., 2023), suggesting that FI effect on morphometric changes on d6 as observed in the current study may be temporary (Marion et al., 2002). Engelsmann et al. (2023) also reported an increased probability for diarrhea (measured by fecal scores), higher antibiotic use, and inflammatory markers in blood for high FI d0 to d4 pigs albeit with their improved growth. However, we did not observe differences in antibiotic use or fecal scores (data not shown). Differences in health status, dietary interventions, or the fact that pigs were individually caged in that study may explain the disagreement between that study and the current one. Although inflammatory markers were not determined in the present study, the increased concentrations of histamine, cadaverine, and putrescine in small intestine digesta from Low FId1-3 pigs suggested GIT microbial dysbiosis with a higher risk for inflammation compared to their High FId1-3 counterparts, which is in line with other research (Kim et al., 2012; Zhang et al., 2020).

Histamine, which can act as a hormone and neurotransmitter, was notably increased (×4.5) in small intestine digesta of Low FId1-3 pigs. Histamine can be produced in the lumen of the gut by microbial decarboxylation of L-histidine or endogenously synthesized by mast cells, enterochromaffin cells, and histaminergic neurons to exert a pro-inflammatory response (Maintz and Novak, 2007; Wang et al., 2018). Therefore, part of the histamine reported in Low FId1-3 pigs is likely endogenous since its increase was greater than the one for other biogenic amines. In fact, histamine is a valid biomarker for gut health in pigs also prior to weaning (Ramsay et al., 2020). In previous studies, elevated histamine levels originating from high protein diets were associated with diarrhea (Pieper et al., 2012) and the induction of Cl^—^secretion (Kröger et al., 2013) in pigs. Although we did not find significant differences in CP content in whole small intestine or colon at d6, a carryover effect of low FI during first days after weaning on protein malabsorption is expected with the reduced intestinal surface observed. As such, the consequences may impact intestinal microbiota, produce biogenic amines and inflammation (Moeser et al., 2017; Li et al., 2022) in agreement with the poor performance observed for Low FId1-3 pigs. Under healthy conditions, histamine and other biogenic amines are detoxified by enzymes in the intestinal epithelium i.e., histamine N-methyltransferase, monoamine, and diamine oxidases (Hughes et al, 2000). Arguably, the hypothetical loss of such enzyme activity in the villi tips may occur due to damage or shortening of the villi which was observed in Low FId1-3. Hence, reduced surface area and enterocyte maturity would further contribute to the negative effects of histamine. Interestingly, Kröger et al. (2013) demonstrated that pigs adapt to such conditions and reported an increased activity and gene expression of histamine-degrading enzymes by 21 d following weaning when fed a high CP diet (20% vs. 14.5% CP). Nonetheless, even after an eventual adaptation, the disruption of nutrient intake and the presence of undigested protein and protein catabolites immediately after weaning seem detrimental to growth and FI as demonstrated in the present study.

## Conclusion

A high FI early after weaning was associated with a greater development of the gastrointestinal tract and reduced protein fermentation in SI digesta at d6 after weaning. Furthermore, such a phenotype appears to be associated with faster eaters and higher FI and growth toward the end of the nursery phase. The effect of weaning BW was additive and independent from FId1-3 with higher weaning BW increasing growth performance from the second half of the nursery. The present study highlighted and reinforced the relevance of weaning BW and initial FI as independent factors influencing feeding patterns and performance over time. In general, it is important to continue investigating the factors affecting the individual and initial FI following weaning due to its relevance for gastrointestinal development, effect on feeding patterns and behavior, and carryover effects on FI and growth performance in later life.

## Supplementary Material

skad419_suppl_Supplementary_Tables_S1

## Abbreviations

ADFI: average daily feed intake
ADG: average daily gain
BW: body weight
CP: crude protein
DM: dry matter
EE: ether extract
EFS: electronic feeding station
FE: feed efficiency
FI: feed intake
GC: goblet cells
GIT: gastrointestinal tract
NE: net energy
SID: standardized ileal digestible
VFA: volatile fatty acids

## References

[CIT0001] Brooks, P. H., and C. H. Tsourgiannis. 2003. Factors affecting the voluntary feed intake of the weaned pig. In: Le Dividich J., J. R. Pluske, and M. W. A. Verstegen, editors. Weaning the pig: concepts and consequences. Wageningen, Netherlands: Wageningen Academic Publishers; p. 81–116

[CIT0002] Bruininx, E. M. A. M., C. M. C. Van Der Peet-Schwering, J. W. Schrama, P. F. G. Vereijken, P. C. Vesseur, H. Everts, L. A. Den Hartog, and A. C. Beynen, 2001. Individually measured feed intake characteristics and growth performance of group-housed weanling pigs: effects of sex, initial body weight, and body weight distribution within groups. J. Anim. Sci. 79:301–308. doi:10.2527/2001.792301x 11219437

[CIT0003] Bruininx, E. M. A. M., M. J. W. Heetkamp, D. Van den Bogaart, C. M. C. Van der Peet-Schwering, A. C. Beynen, H. Everts, L. A. Den Hartog, and J. W. Schrama. 2002. A prolonged photoperiod improves feed intake and energy metabolism of weanling pigs. J. Anim. Sci. 80:1736–1745doi:10.2527/2002.8071736x 12162640

[CIT0004] Bruininx, E. M. A. M., C. M. C. Van Der Peet‐Schwering, J. W. Schrama, L. A. Den Hartog, H. Everts, and A. C. Beynen. 2003. The IVOG® feeding station: a tool for monitoring the individual feed intake of group-housed weanling pigs. J. Anim. Physiol. Anim. Nutr. 85:81–87. doi:10.1046/j.1439-0396.2001.00305.xe 11686776

[CIT0005] Carey, H. V., U. L. Hayden, and K. E. Tucker. 1994. Fasting alters basal and stimulated ion transport in piglet jejunum. Am. J. Physiol. 267:R156–63. doi:10.1152/ajpregu.1994.267.1.R156 8048618

[CIT0006] Deplancke, B., and H. R. Gaskins. 2001. Microbial modulation of innate defense: goblet cells and the intestinal mucus layer. Am. J. Clin. Nutr. 73:1131S–1141S doi:10.1093/ajcn/73.6.1131S 11393191

[CIT0007] Dong, G. Z. and J. R. Pluske. 2007. The low feed intake in newly-weaned pigs: problems and possible solutions. Asian-australas. J. Anim. Sci. 20:440–452 10.5713/ajas.2007.440

[CIT0008] Engelsmann, M. N., T. S. Nielsen, M. S. Hedemann, U. Krogh, and J. V. Nørgaard. 2023. Effect of post-weaning feed intake on performance, intestinal morphology, and the probability of diarrhoea in piglets. Animal. 17:100891. doi:10.1016/j.animal.2023.100891 37453185

[CIT0053] Eissen, J. J., E. Kanis, and J. W. M. Merks. 1998. Algorithms for identifying errors in individual feed intake data of growing pigs in group-housing. Appl. Eng. Agric. 14:667−673

[CIT0009] Fàbrega, E., J., Tibau, J. Soler, J. Fernández, J. Font, D. Carrión, A. Diestre, and X. Manteca. 2003. Feeding patterns, growth performance and carcass traits in group-housed growing-finishing pigs: the effect of terminal sire line, halothane genotype and age. Anim. Sci. 77:11–21. doi:10.1017/S1357729800053601

[CIT0010] Figueroa, J., D. Solà-Oriol, X. Manteca, and J. F. Pérez. 2013. Social learning of feeding behaviour in pigs: effects of neophobia and familiarity with the demonstrator conspecific. Appl. Anim. Behav. Sci. 148:120–127. doi:10.1016/j.applanim.2013.06.002

[CIT0011] Gao, H., G. Su, J. Jensen, P. Madsen, O. F. Christensen, B. Ask, B. G. Poulsen, T. Ostersen, and B. Nielsen. 2021. Genetic parameters and genomic prediction for feed intake recorded at the group and individual level in different production systems for growing pigs. Genet. Sel. 53:1–12. doi:10.1186/s12711-021-00624-3 PMC8028714 33832423

[CIT0012] Hall, A. D., W. G. Hill, P. R. Bampton, and A. J. Webb. 1999. Genetic and phenotypic parameter estimates for feeding pattern and performance test traits in pigs. Anim. Sci. 68:43–48. doi:10.1017/s1357729800050062

[CIT0013] Hughes, R., E. A. Magee, and S. Bingham. 2000. Protein degradation in the large intestine: relevance to colorectal cancer. Curr. Issues Intestinal Microbiol. 1:51–58 11709869

[CIT0014] Hyun, Y. and M. Ellis. 2000. Relationships between feed intake traits, monitored using a computerized feed intake recording system, and growth performance and body composition of group-housed pigs. Asian-Australas. J. Anim. Sci. 13:1717–1725. doi:10.5713/ajas.2000.1717

[CIT0015] Kavlak, A. T. and P. Uimari., P. 2019. Estimation of heritability of feeding behaviour traits and their correlation with production traits in Finnish Yorkshire pigs. J. Anim. Breed. Genet. 136:484–494. doi:10.1111/jbg.12408 31172608

[CIT0016] Kelly, D., J. A. Smyth, and K. J. McCracken. 1991. Digestive development of the early-weaned pig: 1. Effect of continuous nutrient supply on the development of the digestive tract and on changes in digestive enzyme activity during the first week post-weaning. Br. J. Nutr. 65:169–180. doi:10.1079/bjn19910078 1904270

[CIT0017] Kim, J. C., C. F. Hansen, B. P. Mullanand, and J. R. Pluske. 2012. Nutrition and pathology of weaner pigs: nutritional strategies to support barrier function in the gastrointestinal tract. Anim. Feed Sci. Technol. 172, 3–16. doi:10.1016/j.anifeedsci.2011.12.022

[CIT0018] Koenig, J. E., A. Spor, N. Scalfone, A. D. Fricker, J. Stombaugh, R. Knight, L. T. Angenent, and R. E. Ley. 2011. Succession of microbial consortia in the developing infant gut microbiome. Proc. Natl. Acad. Sci. U.S.A. 108:4578–4585. doi:10.1073/pnas.1000081107 20668239 PMC3063592

[CIT0019] Kröger, S., R. Pieper, H. G. Schwelberger, J. Wang, C. Villodre Tudela, J. R. Aschenbach, A. G. Van Kessel, and J. Zentek. 2013. Diets high in heat-treated soybean meal reduce the histamine-induced epithelial response in the colon of weaned piglets and increase epithelial catabolism of histamine. PLoS One. 8:e80612. doi:10.1371/journal.pone.0080612 24260435 PMC3833947

[CIT0020] Lallès, J. P., P. Bosi, H. Smidt, and C. R. Stokes. 2007. Nutritional management of gut health in pigs around weaning. Proc. Nutr. Soc. 66:260–268. doi:10.1017/S0029665107005484 17466106

[CIT0021] Laskoski, F., J. E. G. Faccin, M. L. Bernardi, A. P. G. Mellagi, R. R. Ulguim, G. F. R. Lima, M. A. D. Gonçalves, U. A. D. Orlando, R. Kummer, I. Wentz, and F. P. Bortolozzo. 2021. Effects of different feeder and floor space allowances on growth performance and welfare aspects in nursery pigs. Livest. Sci. 249:104533. doi:10.1016/j.livsci.2021.104533

[CIT0022] Le Dividich, J. and P. Herpine. 1994. Effects of climatic conditions on the performance, metabolism and health status of weaned pigs. Livest. Prod. Sci. 38:79–90.

[CIT0023] Leon, R. M., Borner, T. Reiner, D. J. Stein, L. M. Lhamo, R. De Jonghe, B. C. and M. R. Hayes. 2019. Hypophagia induced by hindbrain serotonin is mediated through central GLP-1 signaling and involves 5-HT2C and 5-HT3 receptor activation. Neuropsychopharmacol. 44:1742–1751. doi:10.1038/s41386-019-0384-x PMC6784912 30959513

[CIT0024] Li, Q. and J. F. Patience. 2017. Factors involved in the regulation of feed and energy intake of pigs. Anim. Feed Sci. Technol. 233, 22–33. 10.1016/j.anifeedsci.2016.01.001

[CIT0025] Li, Z., Ding, L. Zhu, W. and S. Hang. 2022. Effects of the increased protein level in small intestine on the colonic microbiota, inflammation and barrier function in growing pigs. BMC Microbiol. 22:1–11. doi:10.1186/s12866-022-02498-x 35794527 PMC9258065

[CIT0026] López-Vergé, S., J. Gasa, M. Farré, J. Coma, J. Bonet, and D. Solà-Oriol. 2018. Potential risk factors related to pig body weight variability from birth to slaughter in commercial conditions. Transl. Anim. Sci. 2:383–395. doi:10.1093/tas/txy082 32704721 PMC7200415

[CIT0027] Maintz, L., and N. Novak. 2007. Histamine and histamine intolerance. Am. J. Clin. Nutr. 85:1185–96. doi:10.1093/ajcn/85.5.1185 17490952

[CIT0028] Margolis, K. G., J. F. Cryan, and E. A. Mayer. 2021. The microbiota-gut-brain axis: from motility to mood. Gastroenterology. 160:1486–1501. doi:10.1053/j.gastro.2020.10.066 33493503 PMC8634751

[CIT0029] Marion, J., M. Biernat, F. Thomas, G. Savary, Y. Le Breton, R. Zabielski, I. Le Huerou- Luron, and J. Le Dividich. 2002. Small intestine growth and morphometry in piglets weaned at 7 days of age. Effects of level of energy intake. Reprod. Nutr. Dev. 42:339–354. doi:10.1051/rnd:2002030 12510875

[CIT0030] Moeser, A. J., C. S. Pohl, and M. Rajput. 2017. Weaning stress and gastrointestinal barrier development: implications for lifelong gut health in pigs. Anim. Nutr. 3:313–321. doi:10.1016/j.aninu.2017.06.003 29767141 PMC5941262

[CIT0031] Montoro, J. C., E. G. Manzanilla, D. Solà-Oriol, R. Muns, J. Gasa, O. Clear, and J. A. Calderón Díaz. 2020. Predicting productive performance in grow-finisher pigs using birth and weaning body weight. Animals. 10:1017. doi:10.3390/ani10061017 32545432 PMC7341257

[CIT0032] NRC. 2012. Nutrient requirements of swine. 11th rev. ed. Washington, (DC): Natl. Acad. Press.

[CIT0033] Owsley, W. F., D. E. Orr Jr, and L. F. Tribble. 1986. Effects of age and diet on the development of the pancreas and the synthesis and secretion of pancreatic enzymes in the young pig. J. Anim. Sci. 63:497–504. doi:10.2527/jas1986.632497x 2428799

[CIT0034] Pelleymounter, M. A., M. Joppa, M. Carmouche, M. J. Cullen, B. Brown, B. Murphy, D. E. Grigoriadis, N. Ling, and A. C. Foster. 2000. Role of corticotropin-releasing factor (CRF) Receptors in the anorexic syndrome induced by CRF. J. Pharmacol. Exp. Ther. 293:799–806. 10869378

[CIT0035] Pieper, R., S. Kroger, J. F. Richter, J. Wang, L. Martin, J. Bindelle, J. K. Htoo, D. von Smolinski, W. Vahjen, J. Zentek, and A. G. van Kessel. 2012. Fermentable fiber ameliorates fermentable protein-induced changes in microbial ecology, but not the mucosal response, in the colon of piglets. J. Nutr. 142:661–667. doi: 10.3945/jn.111.156190 22357743

[CIT0036] Pluske, J. R., I. H. Williams, and F. X. Aherne. 1996. Maintenance of villous height and crypt depth in piglets by providing continuous nutrition after weaning. Anim. Sci. 62: 131–144. doi: 10.1017/s1357729800014417

[CIT0037] Pluske, J. R., D. J. Hampson, and I. H. Williams. 1997. Factors influencing the structure and function of the small intestine in the weaned pig: a review. Livest. Prod. Sci. 51:215–236. doi: 10.1016/s0301-6226(97)00057-2

[CIT0038] Pluske, J. R., D. J. Kerton, P. D. Cranwell, R. G. Campbell, B. P. Mullan, R. H. King, G. N. Power, S. G. Pierzynowski, B. Westrom, C. Rippe, O. Peulen, and F. R. Dunshea. 2003. Age, sex, and weight at weaning influence organ weight and gastrointestinal development of weanling pigs. Aust. J. Agric. Res. 54:15–527. doi:10.1071/AR02156

[CIT0039] Pluske, J. R., D. W. Miller, S. O. Sterndale, and D. L. Turpin. 2019. Associations between gastrointestinal-tract function and the stress response after weaning in pigs. Anim. Prod. Sci. 59:2015–2022. doi:10.1071/an19279

[CIT0040] Quiniou, N. and J. Noblet. 2012. Effect of the dietary net energy concentration on feed intake and performance of growing-finishing pigs housed individually. J. Anim. Sci. 90:4362–4372. doi:10.2527/jas.2011-4004 22696619

[CIT0041] Ramsay, T. G., S. Kahl, J. A. Long, and K. L. Summers. 2020. Peripheral histamine and neonatal growth performance in swine. Domest. Anim. Endocrinol. 70:106370. doi:10.1016/j.domaniend.2019.06.002 31585314

[CIT0042] Rauw, W. M., J. Soler, J. Tibau, J. Reixach, and L. G. Raya. 2006. The relationship between residual feed intake and feed intake behavior in group-housed Duroc barrows. J. Anim. Sci. 84:956–962. doi:10.2527/2006.844956x 16543574

[CIT0043] Roberts, R. R., J. F. Murphy, H. M. Young, and J. C. Bornstein. 2007. Development of colonic motility in the neonatal mouse-studies using spatiotemporal maps. Am. J. Physiol. Gastrointest. 292:G930–8. doi:10.1152/ajpgi.00444.2006 17158255

[CIT0044] Rosier, J., and C. A. Van Peteghem. 1988. Screening method for the simultaneous determination of putrescine, cadaverine, histamine, spermidine and spermine in fish by means of high pressure liquid chromatography of their 5-dimethylaminonaphthalene-1-sulphonyl derivatives. Z. Lebensm. Unters. Forsch. 186:25–8. doi:10.1007/bf01027175 3354263

[CIT0045] Salfen, B. E., J. A. Carroll, and D. H. Keisler. 2003. Endocrine responses to short-term feed deprivation in weanling pigs. J. Endocrinol. 178:541–551. doi:10.1677/joe.0.1780541 12967345

[CIT0046] Spreeuwenberg, M. A. M., J. M. A. J. Verdonk, H. R. Gaskins, and M. W. A. Verstegen. 2001. Small intestine epithelial barrier function is compromised in pigs with low feed intake at weaning. J. Nutr. 131:1520–1527. doi:10.1093/jn/131.5.1520 11340110

[CIT0047] Tang, T., W. J. J. Gerrits, I. Reimert, C. M. C. van der Peet-Schwering, and N. M. Soede. 2022. Variation in piglet body weight gain and feed intake during a 9-week lactation in a multi-suckling system. Animal. 16:100651. doi:10.1016/j.animal.2022.100651 36219999

[CIT0048] Van Kerschaver, C., D. Turpin, J. Michiels, and J. R. Pluske. 2023. Reducing weaning stress in piglets by pre-weaning socialization and gradual separation from the sow: a review. Animals. 13:1644. doi:10.3390/ani13101644 37238074 PMC10215717

[CIT0052] Van Hees, H. M. J., M. Davids, Dominiek Maes, Sam Millet, S. Possemiers, L. A. Den Hartog, T. A. T. G. Van Kempen, and G. P. J. Janssens. 2019. Dietary fibre enrichment of supplemental feed modulates the development of the intestinal tract in suckling piglets. J. Anim. Sci. Biotechnol. 10: 1-11. Doi:10.1186/s40104-019-0386-x 31636904 PMC6794736

[CIT0049] Wang, C. C., H. Wu, F. H. Lin, R. Gong, F. Xie, Y. Peng, J. Feng, and C. H. Hu. 2018. Sodium butyrate enhances intestinal integrity, inhibits mast cell activation, inflammatory mediator production and JNK signaling pathway in weaned pigs. Innate Immun. 24:40–46. doi:10.1177/1753425917741970 29183244 PMC6830759

[CIT0050] Wensley, M. R., M. D. Tokach, J. C. Woodworth, R. D. Goodband, J. T. Gebhardt, J. M. DeRouchey, and D. McKilligan. 2021. Maintaining continuity of nutrient intake after weaning. II. Review of post-weaning strategies. Transl. Anim. Sci. 5:txab022. doi:10.1093/tas/txab022 34841202 PMC8611789

[CIT0051] Zhang, H., N. V. D. Wielen, B. V. D. Hee, J. Wang, W. Hendriks, and M. Gilbert. 2020. Impact of fermentable protein, by feeding high protein diets, on microbial composition, microbial catabolic activity, gut health and beyond in pigs. Microorganisms. 8:1735. doi:10.3390/microorganisms8111735 33167470 PMC7694525

