# Correction to: Variability in feed intake the first days following weaning impacts gastrointestinal tract development, feeding patterns, and growth performance in nursery pigs

**DOI:** 10.1093/jas/skae072

**Published:** 2024-04-13

This is a correction to: Lluís Fabà, Tetske G Hulshof, Kelly C M Venrooij, Hubèrt M J Van Hees, Variability in feed intake the first days following weaning impacts gastrointestinal tract development, feeding patterns, and growth performance in nursery pigs, *Journal of Animal Science*, Volume 102, 2024, skad419, https://doi.org/10.1093/jas/skad419.

In the originally published version of this manuscript, the wrong numbers were typed for the cumulative feed intake level classes. However, this does not have any implication on the data, data analysis, or result interpretation other than the absolute values of feed intake described and associated to each class in the following locations:

- General classification of pigs. The cumulative feed intake between day 1 and day 3 should be: High FId1-3 = 626 ± 193 g and Low FId1-3 = 311 ± 181 g.
- General classification of pigs feed intake x weaning weight class interaction values should be: High FId1-3 High BW0 = 637; High FId1-3 Low BW0 = 616; Low FId1-3 High BW0 = 291; Low FId1-3 Low BW0 = 332 (Table1).
- For the selected pigs for euthanasia. The cumulative feed intake between day 1 and day 3 classes should be: High FId1-3 = 783 g and Low FId1-3 = 236 g FId1-3. (Table 2)

These errors have been corrected in 5 places: the abstract, material and methods, and results section, and Tables 1 and 2. The authors apologize for any inconvenience this may have caused.

**Table 1.** Effects of feed intake d1-3 class (FId1-3) and weaning body weight class (BW0) on overall nursery performance^1,2^.

		N	FI d1-3, g	BW d0, kg	BW d40, kg	ADG d0-40, g/d	ADFI d0-40, g/d	FE d0-40, g/d
FI d1-3	High	58	626	7.26	24.0	400	573	0.70
	Low	53	311	7.06	21.8	351	512	0.69
	SEM		76.4	0.111	0.37	8.45	11.28	0.011
	P-value		<.0001	0.049	<.0001	<.0001	0.0001	0.287
BW0 class	High	56	464	7.70	24.2	394	565	0.70
	Low	55	474	6.62	21.6	357	520	0.69
	SEM		76.6	0.110	0.36	10.38	10.86	0.012
	P-value		0.377	<.0001	<.0001	<.001	0.004	0.142
FId1-3 × BW0 means								
High FId1-3	High BW0	34	637	7.82	25.1	414	595	0.70
	Low BW0	24	616	6.71	22.9	385	552	0.70
Low FId1-3	High BW0	22	291	7.58	23.3	374	536	0.70
	Low BW0	31	332	6.54	20.3	328	488	0.68

**Table 2.** General traits and gastrointestinal measurements for pigs selected based on feed intake d1-3 (FId1-3)^1^.

	FI d1-3 class			
	High	Low	SEM^2^	P-values
	*N* = 12	*N* = 12		
Body weight dissection, kg	8.32	7.76	0.29	0.135
Body weight weaning, kg	7.05	7.25	0.25	0.525
Cumulative feed intake d1-3, g	783^a^	236^b^	58	<0.001
Average daily feed intake d0-6, g/d	261^a^	124^b^	13.2	<0.001
Average daily gain d0-6, g/d	211^a^	85^b^	14.9	<0.001
Feed intake hours prior euthanasia, g				
2h, g	41.5	37.8	8.96	0.726
8h, g	82.8	79.1	11.5	0.788
12h, g	114	102	16.3	0.529
24h, g	326^a^	251^b^	30.2	0.035
Organ weights, g/kg BW				
Stomach weight	8.37	8.6	0.34	0.503
Stomach content	24.7	29.5	2.99	0.276
Small intestine weight	35.5^a^	28.8^b^	1.28	<0.001
Small intestine content	19.6	17.2	1.70	0.337
Cecum weight	1.68	1.40	0.13	0.159
Cecum content	4.73	4.01	0.68	0.455
Colon + rectum weight	14.9 ^a^	12.9^b^	0.6	0.021
Colon + rectum content	16.4	15.5	1.38	0.599
Pancreas	1.77^a^	1.55 ^b^	0.07	0.028
Liver	26.1	26.0	0.76	0.931

The changes to Tables 1 and 2 are updated below.

