# Supplementary material for: Variability in feed intake the first days following weaning impacts gastrointestinal tract development, feeding patterns, and growth performance in nursery pigs
Source: J Anim Sci. 2023 Dec 23;102:skad419. doi: 10.1093/jas/skad419 (PMC10799316; doi:10.1093/jas/skad419)
Supplement: skad419_suppl_Supplementary_Tables_S1 [file skad419_suppl_supplementary_tables_s1.docx]

**Table S1**. Composition of the diets.

| **Ingredients, %** | **Phase 1 (d0-15)** | **Phase 2 (d15-40)** |
| --- | --- | --- |
| Barley | 16.0 | 10.0 |
| Wheat | 38.0 | 39.6 |
| Maize | 6.87 | 13.1 |
| Whey powder | 4.18 | - |
| Wheat bran | 6.00 | 7.92 |
| Soybean meal | 11.1 | 11.3 |
| Potato protein | 2.42 | 2.29 |
| Soybean oil | 5.47 | 5.56 |
| Diamol^1^ | 3.0 | 3.0 |
| Premix concentrate^2^ | 2.33 | 2.33 |
| Sugar | 1.0 | 1.0 |
| L-Lysine HCl 98% | 0.83 | 0.88 |
| L-Threonine 98% | 0.34 | 0.37 |
| DL-Methionine 99% | 0.28 | 0.29 |
| L-Valine 96.5% | 0.21 | 0.22 |
| Leucine | 0.13 | 0.15 |
| Iso Leucine | 0.08 | 0.10 |
| L-Tryptophan 98% | 0.07 | 0.08 |
| L-Histidine HCl 98% | 0.10 | 0.09 |
| Salt (NaCl) | 0.42 | 0.36 |
| Monocalcium phosphate | 0.88 | 0.98 |
| Sodium Bicarbonate | 0.22 | 0.30 |
| Calcium carbonate | 0.07 | 0.08 |
| **Calculated (and analyzed), g/kg** |  |  |
| Net energy, MJ/kg | 10.6 | 10.6 |
| Moisture | 114 (107) | 119.4 (111) |
| Crude Protein | 170 (171) | 168 (168) |
| Crude Fat (ee) | 71.8 (83) | 74.6 (76) |
| Crude Fiber | 27.8 (31) | 28.4 (30) |
| Ash | 81.1 (76) | 80.2 (76) |
| Insoluble ash | (25.4) | (25.8) |
| Neutral detergent fiber | 103.8 | 106.6 |
| Acid detergent fiber | 38.5 | 40.0 |
| Acid detergent lignin | 7.5 | 7.6 |
| Non starch polysaccharides | 130.0 | 127.3 |
| Lys | 14.2 (13.1) | 14.2 (14.4) |
| SID Lys^3^ | 13.1 | 13.1 |
| SID Met | 5.22 | 5.28 |
| SID Met + Cys | 7.59 | 7.59 |
| SID Trp | 2.36 | 2.36 |
| SID Thr | 8.25 | 8.25 |
| SID Ile | 6.28 | 6.28 |
| SID Arg | 8.20 | 8.25 |
| SID Leu | 11.52 | 11.52 |
| SID Val | 8.51 | 8.51 |
| SID His | 3.67 | 3.67 |
| Na | 2.5 (2.3) | 2.5 (2.4) |
| K | 7.5 | 6.75 |
| Cl | 5.4 | 4.65 |
| dEB | 150 | 150 |
| Ca | 6 (6.3) | 6 (5.9) |
| P | 5.7 (5.5) | 6 (5.5) |
| Zn, mg/kg | 136.8 (131) | 138.5 (126) |

^1^Diamol: added as source of acid insoluble ash for calculating digestibility (Imerys Industrial Minerals Denmark A/S).

^2^Premix concentrate; Supplied per kilogram of complete diet: Vitamin A, 8000 IU; Vitamin D3, 2000 IU; Vitamin E, 30 IU; Vitamin K3-menadione, 1.5 mg; Vitamin B12, 0.03 mg; Thiamine, 1.00 mg; Niacin, 20 mg; Riboflavin, 4 mg; Pantothenate, 13 mg; Folic acid, 0.30 mg; Pyridoxine, 1.0 mg; Iron sulphate, 100 mg; Zinc sulphate, 100 mg; Magnesium oxide, 30 mg; Copper sulphate, 20 mg; Copper chelate, 70 mg; Sodium selenite, 0.30 mg; Iodine, 1 mg; Phytase, 550 FTU; organic acid and medium chain fatty acids blend, 11.5 g/kg.

^3^SID: Standardized ileal digestible.
